# Correlation between anti-mullerian hormone with insulin resistance in polycystic ovarian syndrome: a systematic review and meta-analysis

**DOI:** 10.1186/s13048-024-01436-x

**Published:** 2024-05-18

**Authors:** Mohd Zakwan Md Muslim, Aniza Mohammed Jelani, Noorazliyana Shafii, Najib Majdi Yaacob, Noor Azlin Azraini Che Soh, Hanim Afzan Ibrahim

**Affiliations:** 1 Department of Chemical Pathology, School of Medical Sciences, Health Campus, Universiti Sains Malaysia, Kelantan, 16150 Malaysia; 2 Unit of Biostatistics and Research Methodology, School of Medical Sciences, Health Campus, Universiti Sains Malaysia, Kelantan, 16150 Malaysia; 3 School of Dental Sciences, Health Campus, Universiti Sains Malaysia, Kelantan, 16150 Malaysia

**Keywords:** anti-Müllerian hormone, Polycystic ovary syndrome, Insulin resistance, Homeostatic model assessment for insulin resistance, Systematic review, Meta-analysis

## Abstract

**Background:**

Epidemiological studies regarding the correlation between anti-Müllerian hormone (AMH) and insulin resistance (IR) in polycystic ovarian syndrome (PCOS) remain inconsistent. The primary aim of this study was to determine the correlations between AMH and IR in patients with PCOS and to explore the selected factors that influence the correlations.

**Methods:**

We conducted systemic searches of online databases (PubMed, Science Direct, Taylor and Francis, Scopus, and ProQuest) from inception to December 20, 2023 and manual searches of the associated bibliographies to identify relevant studies. We then performed subgroup and sensitivity analyses to explore the sources of heterogeneity, followed by a publication bias risk assessment of the included studies using the Joanna Briggs Institute critical appraisal tool. We used a random-effects model to estimate the pooled correlations between AMH and the homeostatic model assessment for insulin resistance (HOMA-IR) in patients with polycystic ovarian syndrome (PCOS).

**Results:**

Of the 4835 articles identified, 22 eligible relevant studies from three regions were included and identified as low risk of bias. The random-effects pooled correlation estimate was 0.089 (95% confidence interval [CI]: −0.040, 0.215), with substantial heterogeneity (I^2^ = 87%; τ^2^ = 0.0475, *p* < .001). Subgroup analyses showed that the study region did not influence the correlation estimates, and sensitivity analysis showed no significant alteration in the pooled correlation estimate or 95% CI values. No publication bias was observed.

**Conclusion:**

There was a weak, statistically insignificant correlation between AMH and HOMA-IR in patients with PCOS. The correlation estimates did not vary according to the study participants’ regions.

## Background

Polycystic ovarian syndrome (PCOS) is a highly prevalent endocrine disorder that affects 4–20% of reproductive-age women globally [1]. Metabolism plays an important role in the long-term sequelae of the condition. Central obesity, decreased glucose tolerance, and/or dyslipidemia are the most frequent metabolic abnormalities in PCOS, all of which are related to insulin resistance (IR) [2].

IR is a condition in which target organs fail to respond properly to insulin. It is a common metabolic derangement occurring in PCOS and is seen in all of the disease phenotypes [3]. Epigenetic changes such as DNA methylation, histone status, and miRNA expression are among the several factors that are hypothesized to play a role in the development of IR in PCOS patients [3]. Apart from that, environmental factors, dietary changes, inflammation, and vitamin D deficiency can also have an impact on insulin sensitivity in these patients [3]. IR leads to compensatory hyperinsulinemia, which stimulates the transcription of the gonadotropin-releasing hormone gene in the hypothalamus. As a result, there is an increase in luteinizing hormone pulse frequency at the hypophysis, which subsequently elevates androgen synthesis by the ovary. Hyperinsulinemia also increases androgenic production by directly stimulating the ovary to produce androgens and decreasing sex hormone–binding globulin synthesis by the liver. Hyperandrogenism may in turn worsen IR, creating a vicious cycle of IR–hyperinsulinemia–hyperandrogenemia in PCOS [3–6].

Anti-Müllerian hormone (AMH), also known as Müllerian-inhibiting substance, is a 140-kDa dimeric glycoprotein that belongs to transforming growth factor–β family [7]. It is secreted by the granulosa cells of growing ovarian follicles from the primary to small antral stages [7]. The hormone is well known for its role as a marker of ovarian reserve [8], and its potential role as a surrogate marker for the diagnosis of PCOS [9, 10]. AMH is thought to play an important role in the etiology of the disease because it can inhibit the formation of primary follicles and their recruitment, contributing to follicular arrest [11].

Many studies have investigated the correlation between AMH and IR in PCOS, but the results have been inconsistent. Despite an increasing number of intervention studies assessing the impact of AMH and IR on PCOS, there remains a lack of solid data indicating a causal relationship between AMH and IR. Although several studies have found a strong positive association between AMH and IR, others have found a negative correlation. Thus, the objective of this systematic review and meta-analysis (SRMA) was to quantitatively summarize the current evidence to determine whether levels of AMH correlate with IR in PCOS. Knowledge of the relationship between AMH and IR may contribute to a better understanding of the pathophysiology of PCOS and its metabolic complications. Furthermore, the finding of a significant correlation would increase the plausibility of a biological link between AMH and IR in PCOS and suggest a potential avenue for PCOS treatment. To the best of our knowledge, this is the first SRMA to investigate the relationship between AMH and IR in PCOS.

## Materials and methods

### Design and protocol development

The protocol for this SRMA was registered in the International Prospective Register of Systematic Reviews (PROSPERO; registration No. CRD42021255383; Appendix A). We used the Preferred Reporting Items for Systematic Reviews and Meta-analysis (PRISMA) guidelines to report the SRMA results [12].

### Eligibility criteria

Two investigators (M.Z.M.M. and A.M.J.) independently screened all titles and abstracts from the initial search and full-text articles identified during the first-stage screening. We included studies from inception to December 20, 2023, reporting primary data for Pearson’s correlations between AMH and homeostatic model assessment for insulin resistance (HOMA-IR) in PCOS. The searches were conducted in English, and only articles published in English were selected. Observational studies, such as cross-sectional, cohort, or longitudinal studies, were eligible for inclusion if they reported target populations of reproductive-age women diagnosed with PCOS according to the Rotterdam criteria. We excluded experimental (randomized and nonrandomized) trials, case reports, ecological studies, case reports, studies that did not involve human participants (animal and in vitro studies), book chapters, narrative reviews, and protocol studies.

### Data source and search strategy

Two investigators (M.Z.M.M. and A.M.J.) extensively searched online international databases to which our institutional library subscribed (PubMed, ScienceDirect, Taylor and Francis, Scopus, and ProQuest) from inception to December 20, 2023. We used the following MeSH terms and text words linked to AMH, HOMA-IR, and PCOS: “Müllerian-inhibiting factor,” “anti Müllerian hormone,” “Müllerian-inhibitory substance,” “resistance, insulin,” “insulin sensitivity,” “homeostatic model assessment for insulin resistance,” “insulin resistance,” “Müllerian regression factor,” “ovary syndrome, polycystic,” “syndrome, polycystic ovary,” “Stein–Leventhal syndrome,” “polycystic ovarian syndrome,” “sclerocystic ovarian degeneration,” and “sclerocystic ovary.” We tested the search strategy in PubMed and further refined it based on its efficacy in retrieving relevant studies from each database. To identify other relevant research, we conducted forward and backward reference chaining of the included studies and searched the reference lists of the included papers. We applied an OR Boolean operator to connect all MeSH terms to maximize the sensitivity of the literature search (Appendix B).

### Selection process

Two reviewers (M.Z.M.M. and A.M.J.) conducted an independent preliminary screening of the titles, abstracts, and selected articles that potentially met the inclusion criteria. We used Microsoft Excel 365 to sort the data and then retrieved and reassessed the full texts that met the eligibility criteria. To avoid bias in the study selection, we conducted the eligibility assessment in duplicate and independently. Discrepancies were resolved through discussion and consensus between the reviewers and the third author (N.S.). All three authors were in complete agreement with the final decision and documented detailed reasons for the exclusion of sources.

### Data extraction

We downloaded the search results from each database and then imported them into the Zotero software using the Zotero web connector. We removed duplicate articles using Zotero software, exported the search results in Microsoft Excel.csv format, and later converted them to .xlsx format.

Two reviewers (M.Z.M.M. and A.M.J.) conducted a preliminary screening of titles and abstracts to identify potential articles of interest. The full texts of the potentially eligible studies were retrieved and reassessed according to the inclusion/exclusion criteria. To avoid bias in the study selection process, the reviewers independently assessed eligibility in duplicate and resolved conflicts regarding study identification through discussion with the third researcher (N.S.) to reach 100% agreement on the final decision. We prepared a detailed report explaining why studies were excluded following the full-text review.

After the studies were identified, two investigators (M.Z.M.M. and A.M.J.) abstracted data from the included studies using a standardized predesign and prepiloted electronic data abstraction Microsoft Excel form to assess the study quality and synthesize the evidence. We conducted data abstraction independently to minimize the risk of errors. The abstracted information included the author’s name, publication year, country, region, study design, study subjects, PCOS criteria used, method/platform for AMH measurement, AMH value, HOMA-IR value, and Pearson’s correlation coefficient (*r*) for AMH and HOMA-IR in PCOS.

In cases in which there were multiple publications of the same study, we extracted the most complete and up-to-date data from each publication. We then analyzed the data after eliminating overlaps in the extracted data. We report the literature search and screening outputs using a PRISMA flow diagram.

### Methodological quality assessment

Two authors (M.Z.M.M. and A.M.J.) independently performed the quality assessment using the Joanna Briggs Institute (JBI) Critical Appraisal for Cross-Sectional Studies [13] checklist, which consists of eight questions for assessing specific domains of cross-sectional studies to determine the potential risk of bias; questions can be answered with “yes,” “no,” “unclear,” or “not applicable” (Appendix C). We resolved any disagreement through discussion with the third review author (N.S.). Finally, we summed the scores and converted them to percentages. We classified the risk of bias in each study as high (scores > 50%), moderate (50–69%), or low (≥ 70%) [14]. We included only low-risk studies in this SRMA.

### Data synthesis and statistical analysis

We summarized the descriptions of the original studies using tables and forest plots based on oligo-ovulatory and anovulatory subjects according to Zhang et al. [15]. We entered the data into a Microsoft Excel file before we performed statistical analysis using the Rstudio metacor package [16] (version February 2, 2022) in R (version 4.1.3) [17].

Before pooling the correlation estimates using the inverse variance method, we applied Fisher z-transformations to the correlations. We considered a random-effects model the most appropriate method for computing the summary effect size in the presence of heterogeneity. Therefore, we used a random-effects model with Hartung–Knapp adjustment to estimate the pooled correlation with a 95% confidence interval (CI).

### Heterogeneity assessment

To determine the heterogeneity among the included studies, we used forest plots, tau-squared (τ^2^), Higgins I-squared (I^2^), and Cochrane’s Q test *p* values [18]. We used Schmidt–Hunter estimation to estimate the τ^2^ values and the τ^2^ CIs using the Q-profile method. The τ^2^ and *p* values from the Cochrane’s Q test revealed only the presence versus absence of heterogeneity but did not explain the extent of heterogeneity [19]. We interpreted the τ^2^ values by their CIs, and the Cochrane’s Q test explained the significance of the *p* values. If the τ^2^ CI did not contain zero and the *p* value from the Cochrane’s Q test was significant (*p* < .001), some between-study heterogeneity existed [20]. The amount of heterogeneity in the meta-analysis can be estimated using I^2^. An I^2^ value less than 25% indicates low heterogeneity, a value of 25–75% indicates moderate heterogeneity, and a value of 75% or higher indicates substantial heterogeneity [21].

### Subgroup and sensitivity analysis

To explore the possible causes of heterogeneity, we also conducted subgroup analyses according to region. The random-effects pooled correlation estimate corresponded to the 95% CI, and we reported the within-group and between-group heterogeneity. A *p* value for this test of < 0.10 indicated a statistically significant subgroup effect. We performed a sensitivity analysis using the leave-one-out method to assess the impact of each study on the pooled results by removing one study at a time from the analysis. We used Egger’s test, Begg’s test, and visual inspection of the symmetry in the funnel plots to evaluate publication bias. The level of significance was set at *p* < .05 for the Egger’s and Begg’s tests [22].

## Results

### Study selection

**Fig. 1:**
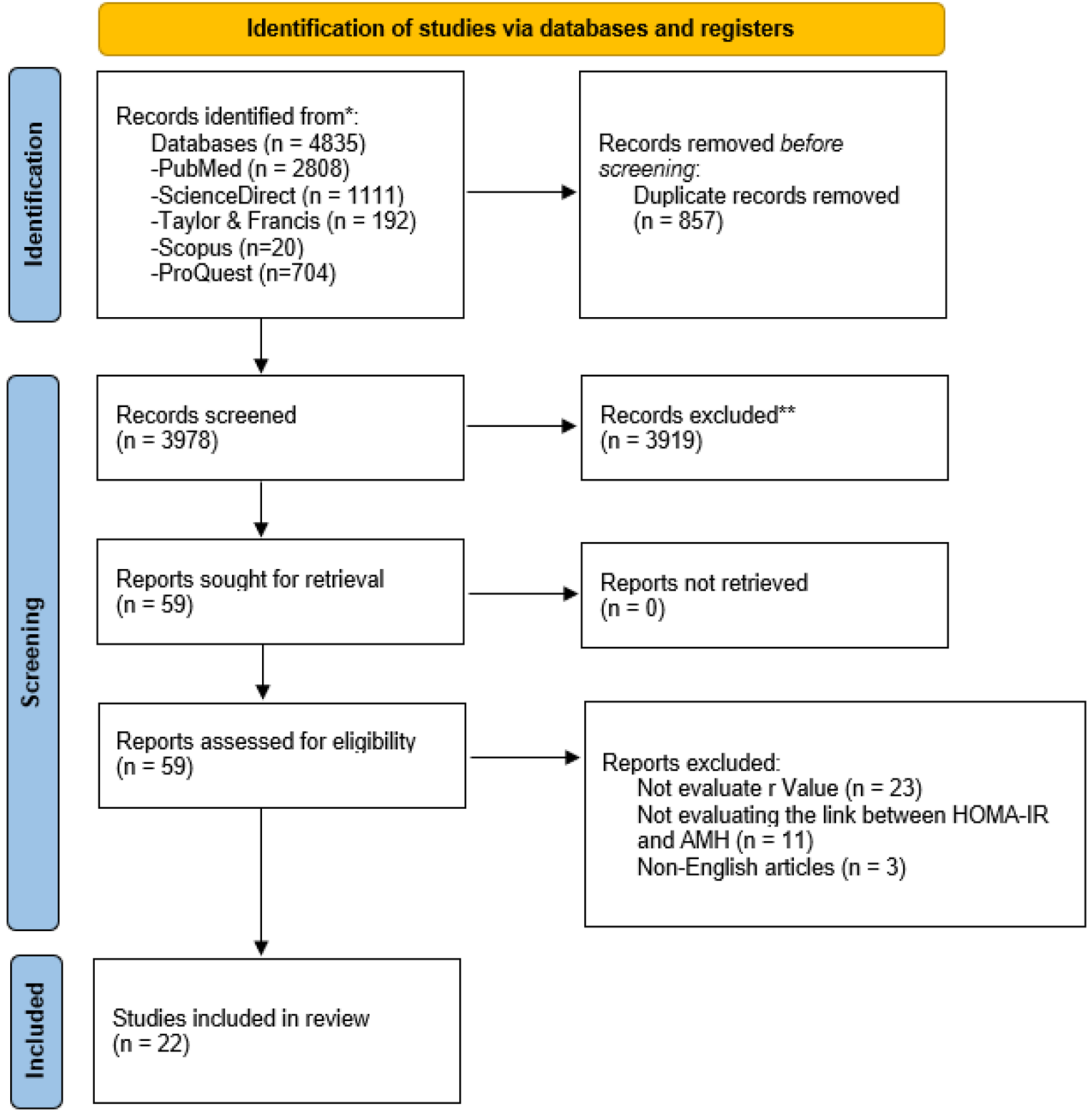
PRISMA flowchart of the review process

We identified 4835 articles through electronic databases and manual searches. After removing duplicates, we screened 3978 titles and abstracts for relevance, yielding 59 full-text articles. After screening the full text of 59 articles, we rejected 37 studies with incorrect statistical data, studies that were irrelevant to this review, and non-English articles. The SMRA covered a final sample of 22 studies (Fig. 1).

### Study characteristics

Table 1 describes the characteristics of the included studies. The studies were published between 2004 and 2023 in 11 countries across three regions, and they included 3,028 PCOS patients. The largest proportions of studies came from Asia (13 studies, 59.1%), Europe (8 studies, 36.4%), and North America (1 study, 4.5%). Most studies (18) were cross-sectional studies, and the remaining four studies were case-control studies. The sample sizes ranged from 12 to 293 PCOS subjects of reproductive age.

**Table 1:** Characteristic of the studies

Authors	Year	Country	Region	Design	Study subject	Age (Years)	BMI (kg/m^2^)	PCOS Criteria	Method (AMH Platform)	AMH Value	HOMA-IR	Pearson’s *r* (*p*-value)
Shen et al. [23]	2015	Taiwan	Asia	Case control	165	Mean: 27 +/-5.7	Mean: 24.3 (+/-5.7)	Rotterdam	Not stated	Mean: 9.7 ng/ml (±5.7)	3.47 (±3.71)	-0.0067 -0.223
Feldman et al. [24]	2017	United States	North American	Cross-sectional	252	Mean: 28.4+/-5.6	Mean: 33(+/-9.5)	Rotterdam	ELISA (Beckmann Coulter)	Median: 5.1 ng/ml (IQR: 3.0–8.1)	Not Stated	-0.3 (< 0.001)
La Marca et al. [25]	2004	Italy	Europe	Cross-sectional	14	Mean: 23	Mean: 25.1	Rotterdam	ELISA (Immunotech)	Mean: 5.0 ng/ml (±1.8)	Not Stated	0.621 (< 0.05)
Chun et al. [26]	2015	Korea	Asia	Case control	Group 1: 53	Mean: 26.38 +/- 4.96	Mean: 21.78 (+/-4.13)	Rotterdam	ELISA (Beckmann Coulter)	Group 1: AMH < 10ng/ml = Mean: 7.31 ng/ml (±1.48)	Group 1: AMH < 10ng/ml = 2.36 (±4.53)	0.121
					Group 2: 42	Mean: 27.55 +/-5.78	Mean: 22.62 (+/-7.18)			Group 2: AMH >10ng/ml = Mean: 14.72 ng/ml (±5.91)	Group 2: AMH > 10ng/ml = 3.07 (±6.16)	-0.279
Caglar et al. [27]	2013	Turkey	Europe	Case control	34	Mean: 26+/-2.8	Mean: 22.1 (+/-1.9)	Rotterdam	ELISA (Beckmann Coulter)	Median: 4.17 ng/ml (IQR: 1.9–26.1)	2 (IQR: 60.7–574.0)	-0.01 -0.943
Sahmay et al. [28]	2018	Turkey	Europe	Cross-sectional	Phenotype 1 (PCOM+OA+HA+): 204	Median: 22 (19-26)	Median: 29.35 (25-32)	Rotterdam	ELISA (DSL)	Phenotype 1 (PCOM + OA + HA+): Median: 7.64 ng/ml (IQR: 4.89–12.5)	Phenotype 1 (PCOM + OA + HA+): 2.37. (IQR: 1.58–3.69)	-0.03 -0.63
					Phenotype 2 (PCOM + OA – HA+): 51					Phenotype 2 (PCOM + OA – HA+): Median: 4.99 ng/ml (IQR: 3.11–8.73)	Phenotype 2 (PCOM + OA – HA+): 1.97 (IQR: 1.51–2.57)	
					Phenotype 3 (PCOM + OA + HA-): 15					Phenotype 3 (PCOM + OA + HA -): Median: 6.88 ng/ml (IQR: 4.1–16.09)	Phenotype 3 (PCOM + OA + HA-): 1.95 (IQR: 1.29–3.1)	
					Phenotype 4 (PCOM – OA + HA +): 16					Phenotype 4 (PCOM – OA + HA+): Median: 2.56 ng/ml (IQR: 1.24–3.51)	Phenotype 4 (PCOM – OA + HA+): 2.86 (IQR: 2.12–3.44)	
Tokmak et al. [29]	2016	Turkey	Europe	Cross-sectional	45	Mean: 18.5 +/-2.4	Mean: 22.3 (+/-1.95)	Rotterdam	ELISA (Chromate-4300)	Mean: 10.8 ng/ml (±4.2)	6.1 (±2.5)	0.44 -0.003
Skalba et al. [30]	2011	Poland	Europe	Cross-sectional	87	Mean: 24.8+/-4.1	Mean: 24.1 (+/-4.7)	Rotterdam	ELISA (Immunotech)	Mean: 10.2 ng/ml (±3.7)	2.0 (±1.3)	0.31 (< 0.001)
Jun et al. [31]	2020	Malaysia	Asia	Cross-sectional	30	Mean: 27.8+/-4.08	Mean: 31.2 (+/-6.22)	Rotterdam	ECLIA (Roche Diagnostics)	Median: 6.8 ng/ml (IQR: 2.5–17.9)	5 (IQR: 1.1–19.5)	-0.49 -0.006
Tian et al. [32]	2014	China	Asia	Case control	Phenotype 1 (PCOM + OA + HA+): 40	Mean: 27.90 +/-4.14	Mean: 21.73(+/-2.31)	Rotterdam	ELISA (DSL)	Phenotype 1 (PCOM + OA + HA+): Mean: 8.33 ng/ml (±2.16)	Phenotype 1 (PCOM + OA + HA+): 2.43 (±1.62)	-0.038 -0.592
					Phenotype 2 (PCOM + OA – HA+): 40	Mean: 28.38 +/-3.21	Mean: 21.14 (+/-2.19)			Phenotype 2 (PCOM + OA – HA+): Mean: 5.49 ng/ml (±1.52)	Phenotype 2 (PCOM + OA – HA+): 2.33 (± 1.52)	
					Phenotype 3 (PCOM + OA + HA-): 40	Mean: 29.35 +/- 3.87	Mean: 21.43 (+/-2.19)			Phenotype 3 (PCOM + OA + HA-): Mean: 6.70 ng/ml (±1.19)	Phenotype 3 (PCOM + OA + HA-): 1.96 ( ± 1.66)	
					Phenotype 4 (PCOM – OA + HA+): 40	Mean: 28.60 +/-3.51	Mean: 21.55 (+/-2.20)			Phenotype 4 (PCOM – OA + HA+): Mean: 4.29 ng/ml (±1.22)	Phenotype 4 (PCOM – OA + HA+): 1.85 ( ± 0.88)	
Chen et al. [33]	2008	Taiwan	Asia	Cross-sectional	99	Median: 26 (21-35)	Median: 23.05(17.61-37.11)	Rotterdam	ELISA (Immunotech)	Median: 94.67 pM (IQR: 34.54–237.58)	1.87 (IQR: 0.44–11.72)	-0.22 -0.03
Wiweko et al. [34]	2018	Indonesia	Asia	Cross-sectional	Phenotype 1 (PCOM + OA + HA+): 39	Median: 29 (20-39)	Median:25.5 (+/-4.8)	Rotterdam	ELISA (Beckmann Coulter)	Phenotype 1 (PCOM + OA + HA+): Median: 11.7 ng/ml (IQR: 4.5–23.8)	Phenotype 1 (PCOM + OA + HA+): 4.2 (IQR: 0.5–8.2)	0.52 (< 0.001)
					Phenotype 2 (PCOM + OA-HA+): 26	Median: 32 (23-39)	Median: 25.7 (+/-5.0)			Phenotype 2 (PCOM + OA – HA+): Median: 7.5 ng/ml (IQR: 4.6–23.8)	Phenotype 2 (PCOM + OA – HA+): 2.9 (IQR: 0.6–8.5)	
					Phenotype 3 (PCOM+OA+HA-) 33	Median: 28 (21-36)	Median:26.0 (+/-5.7)			Phenotype 3 (PCOM + OA + HA-): Median: 7.1 ng/ml (IQR: 3.1–9.6)	Phenotype 3 (PCOM + OA + HA-): 2.1 (IQR: 0.4–4.9)	
					Phenotype 4 (PCOM-OA+HA+) 27	Median: 29 (19-35)	Median: 25.8 (+/-3.5)			Phenotype 4 (PCOM – OA + HA+): Median: 9.9 ng/ml (IQR: 4.6–19.5)	Phenotype 4 (PCOM – OA + HA+): 3.2 (IQR: 0.7–8.1)	
Öztürk et al. [35]	2019	Turkey	Europe	Case control	44	Mean: 31.11 +/-3.41	Mean: 25.92(+/-4.57)	Rotterdam	ELISA (SunRed)	Median: 4.1 ng/ml (IQR: 3.2–9.8)	2.55 (IQR: 0.4–7.09)	0.123 (Not stated)
Yetim Şahin et al. [36]	2019	Turkey	Europe	Cross-sectional	Group 1 (Non-obese): 23	Mean: 16.84 +/-1.36		Rotterdam	ELISA (Beckmann Coulter)	Group 1 (non-obese): Mean: 12.36 ng/ml (±9.06)	Group 1 (Non-Obese): 2.04 (±1.16)	-0.066
					Group 2 (Obese): 29	Mean: 16.64 +/-1.48				Group 2 (obese): Mean: 18.07 ng/ml (±13.14)	Group 2 (Obese): 4.35 (±3.00)	-0.641
Gupta et al. [37]	2019	India	Asia	Cross-sectional	150	Mean: 28.2 +/-3.49	Mean: 24.43 (+/-3.82)	Rotterdam	ELISA (Beckmann Coulter)	Median: 9.9 ng/ml (IQR: 7.12–14.4)	2.55 (IQR: 1.91–3.42)	0.005 -0.943
Zhang et al. [15]	2017a	China	Asia	Cross-sectional	22	Mean: 29.09+/-3.18	Mean: 26.35(+/-2.88)	Rotterdam	ELISA (Beckmann Coulter)	Group 1: Oligo-ovulatory *(D2–3 of the menstrual cycle)*: 7.46 ng/ml (IQR: 5.23–9.92)	5.08 (±2.58)	0.108 -0.632
										Group 2: Oligo-ovulatory *(At selection of the dominant follicle)*: 6.30 ng/ml (IQR: 4.31–11.75)		
										Group 3: Oligo-ovulatory *(At the time of mature follicle)*: 6.77 ng/ml (IQR: 5.25–9.64)		
Zhang et al. [15]	2017b	China	Asia	Cross-sectional	12	Mean: 29.83+/-2.48	Mean: 31.24 (+/-4.89)	Rotterdam	ELISA (Beckmann Coulter)	Group 1 Anovulatory *(On D2–3 of the menstrual cycle)*: 16.8 ng/ml (IQR: 11.49–19.74)	9.28 (±4.33)	0.061 -0.85
										Group 2: Anovulatory *(At 60 days or so in the menstrual cycle.)* : 19.96ng/ml (IQR: 17.98–21.93)		
Sharma et al. [38]	2019	India	Asia	Cross-sectional	40	Mean: 23.28+/-4.8	Mean: 25.6 (+/-3.8)	Rotterdam	ECLIA (Roche Diagnostics)	Mean: 9.43 ng/ml (±9.50)	4.72 (±7.58)	0.474 -0.002
Fu et al. [39]	2020	China	Asia	Cross-sectional	30	Mean: 27.64+/-2.3	Mean: 35.08(+/-2.3)	Rotterdam	ELISA (MyBioSource)	Mean: 12.90 ng/ml (±3.3)	6.1 (±2.49)	0.581 0
Sova et al. [40]	2019	Finland	Europe	Cross-sectional	319	Mean: 28.1+/-4.3	Mean:27.3(+/-6.3)	Rotterdam	ELISA (Beckmann Coulter)	Mean: 66.1 pmol/L (±47.4)	2.6 (±2.8)	-0.26 (< 0.001)
Woo et al. [41]	2012	Korea	Asia	Cross-sectional	87	Mean: 25.3+/-5.0	Mean:21.3(+/-3.4)	Rotterdam	ELISA (Immunotech)	Mean: 11.58 ng/ml (±6.31)	3.51 (IQR: 0.95–29.27)	0.092 -0.396
Chao-Yan et al. [42]	2018	China	Asia	Cross-sectional	653	Mean: 26.9+/-4.2	Mean: 26.2 (+/-5.2)	Rotterdam	ELISA (Union Analyzer)	Mean: 9.3 ng/ml (±3.1)	3 (±2.4)	0.1 -0.15
Han Zhao et al. [43]	2023	China	Asia	Retrospective	220	Mean: 28.13 (+/-4.29)	Mean: 27.21 (+/-4.85)	Rotterdam	ELIA (Beckmann Coulter)	Mean: 7.97 (+/-5.10)	4.31 (+/-3.10)	0.223 (<0.01)

All involved studies used the Rotterdam criteria to diagnose PCOS. Most of the selected studies measured AMH levels using the enzyme-linked immunosorbent assay method, and only two studies measured serum AMH levels using the electrochemiluminescence immunoassay method. We assessed IR according to the HOMA-IR method, and we conducted the correlation analyses using Pearson’s correlation coefficient (*r*) for all included studies. Among the studies, one used oligo-ovulatory and anovulatory subjects. Zhang et al. divided the subjects into oligo-ovulatory and anovulatory subjects [15].

### Quality assessment

We assessed the quality of the articles using the JBI checklist.^43^ Each question was applied to each of the 22 articles, and the answer to each question was given as “yes” or “no.” The overall risk is specified at the bottom of Table 2, with the scores summed as percentages. All included studies achieved a > 50% score and were identified as having a moderate to low risk of bias. Two authors (M.Z.M.M. and A.M.J.) independently evaluated the risk and quality of each study, and any confusion was resolved through a consensus team meeting.

**Table 2:** Bias risk assessment

Question	Authors																					
	Shen *et.al*	Feldman *et.al*	La Marca *et.al*	Chun *et al.*	Caglar *et al.*	Sahmay *et al.*	Tokmak *et al.*	Skalba *et al.*	Jun *et al.*	Tian *et al.*	Chen *et al.*	Wiweko *et al.*	Öztürk *et al.*	Yetim Şahin *et al.*	Gupta *et al.*	Zhang *et al.*	Sharma *et al.*	Fu *et al.*	Sova *et al.*	Woo *et al.*	Chao-Yan *et al.*	Han Zhao *et al*
Were the criteria for inclusion in the sample clearly defined?	Yes	Yes	Yes	Yes	Yes	Yes	Yes	No	Yes	Yes	Yes	Yes	Yes	Yes	Yes	Yes	Yes	Yes	Yes	Yes	Yes	Yes
Were the study subjects and the setting described in detail?	Yes	Yes	No	Yes	Yes	Yes	Yes	Yes	Yes	Yes	No	Yes	No	Yes	Yes	No	Yes	No	Yes	Yes	Yes	Yes
Was the exposure measured in a valid and reliable way?	No	No	No	No	No	No	No	No	No	No	No	No	No	No	No	No	No	No	No	No	No	No
Were objective, standard criteria used for measurement of the condition?	Yes	Yes	Yes	Yes	Yes	Yes	Yes	Yes	Yes	Yes	Yes	Yes	Yes	Yes	Yes	Yes	Yes	Yes	Yes	Yes	Yes	Yes
Were confounding factors identified?	Yes	No	Yes	Yes	Yes	Yes	Yes	Yes	Yes	Yes	Yes	No	No	No	No	Yes	Yes	Yes	No	Yes	Yes	Yes
Were strategies to deal with confounding factor stated?	Yes	No	Yes	Yes	Yes	Yes	Yes	Yes	Yes	Yes	Yes	No	No	No	No	Yes	Yes	Yes	No	Yes	Yes	Yes
Were the outcomes measured in a valid and reliable way?	Yes	Yes	Yes	Yes	Yes	Yes	Yes	Yes	Yes	Yes	Yes	Yes	Yes	Yes	Yes	Yes	Yes	Yes	Yes	Yes	Yes	Yes
Was appropriate statistical analysis used?	Yes	Yes	Yes	Yes	Yes	Yes	Yes	Yes	Yes	Yes	Yes	Yes	Yes	Yes	Yes	Yes	Yes	Yes	Yes	Yes	Yes	Yes
Grading	87.5%	62.5%	75%	87.5%	87.5%	87.5%	87.5%	75%	87.5%	87.5%	75%	62,5%	62.5%	62.5%	62.5%	75%	87.5%	75%	62.5%	87.5%	87.5%	87.5%
Risk of Bias	Low	Moderate	Low	Low	Low	Low	Low	Low	Low	Low	Low	Moderate	Moderate	Moderate	Moderate	Low	Low	Low	Moderate	Low	Low	Low

### Meta-analysis correlation of AMH in PCOS and IR

**Fig. 2:**
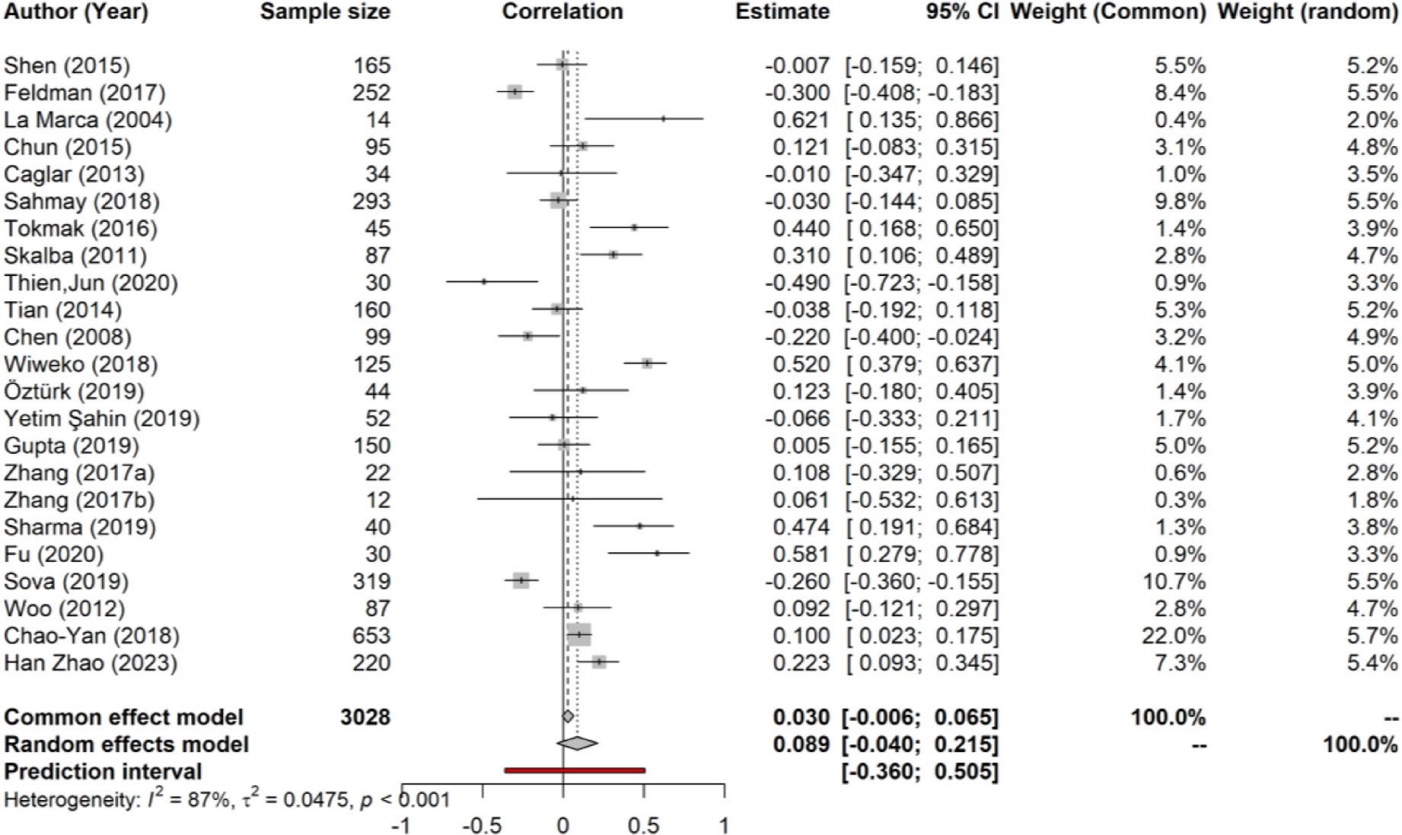
Forest plot of the meta-analysis for the correlations between AMH and IR in PCOS patients

Substantial statistical heterogeneity existed among the individual study estimates (I^2^ = 87%; τ^2^ = 0.0475, *p* < .001). Therefore, we used a random-effects model for the meta-analysis. The overall correlation estimate was 0.089 (95% CI: −0.040, 0.215), which we considered to be a weak correlation (Fig. 2).

### Subgroup and sensitivity analyses

**Fig. 3:**
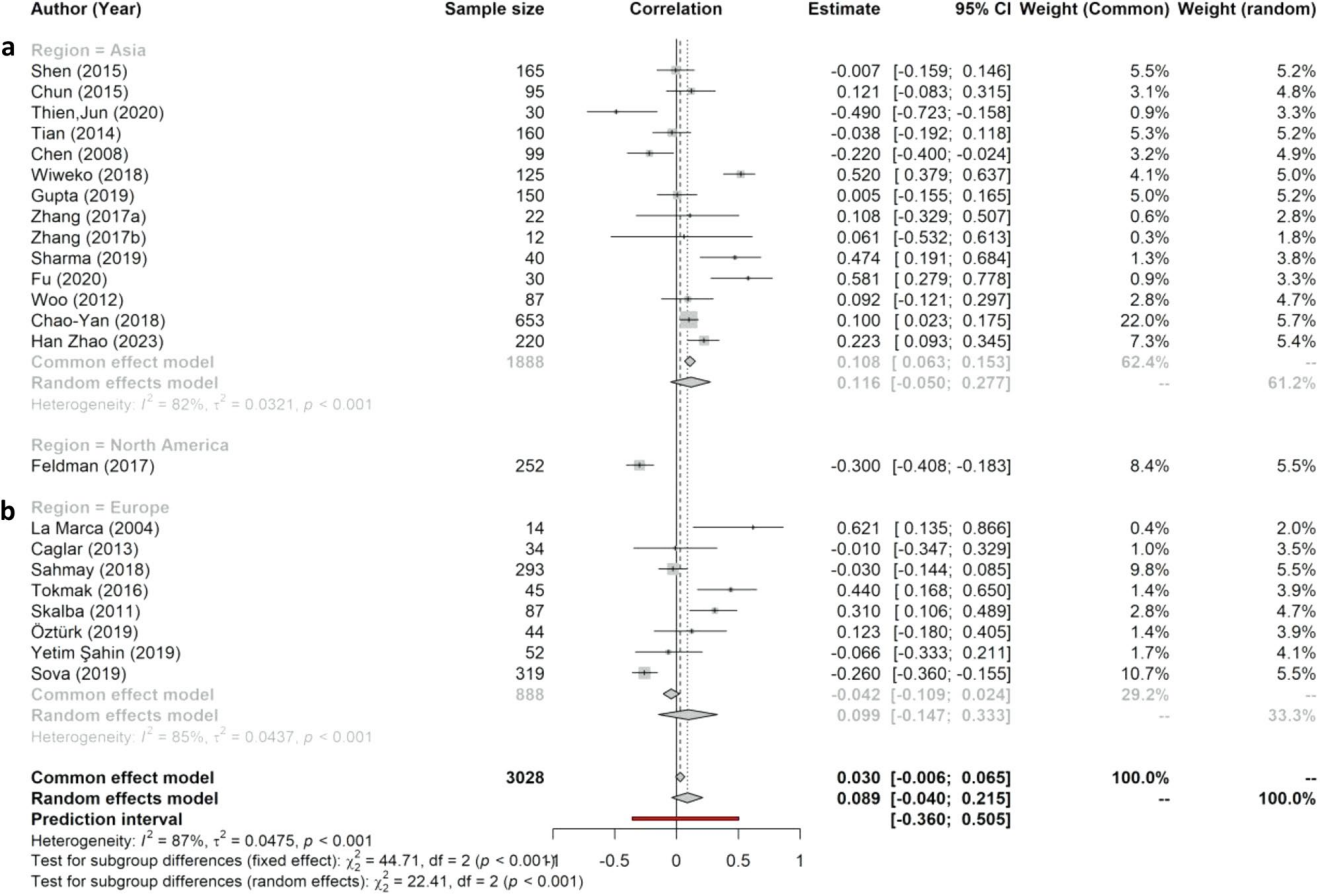
Subgroup analyses based on geographic region: a = Asia, b = Europe

To identify the sources of heterogeneity among the studies, we performed subgroup and sensitivity analyses. Different races and ethnicities may contribute to variations in AMH and IR due to various genetic and environmental factors [44]. The pooled correlation between AMH and HOMA-IR in PCOS patients in Europe [0.099 (95% CI: -0.147, 0.333)] was slightly lower than that in Asia [0.116 (95% CI: -0.050, 0.277]; Fig. 3). The heterogeneity was significant in these two regions: (I^2^ = 85%; τ^2^ = 0.0437, *p* < .001) and (I^2^ = 82%; τ^2^ = 0.0321, *p* < .001), respectively. Although the heterogeneity is significant in these two regions, the pooled correlation did not cause significant variation in this study. Subgroup analyses for other types of possible heterogeneity, such as body mass index (BMI), weight, PCOS phenotype, and age, could not be performed because of inadequate studies and data.

**Fig. 4:**
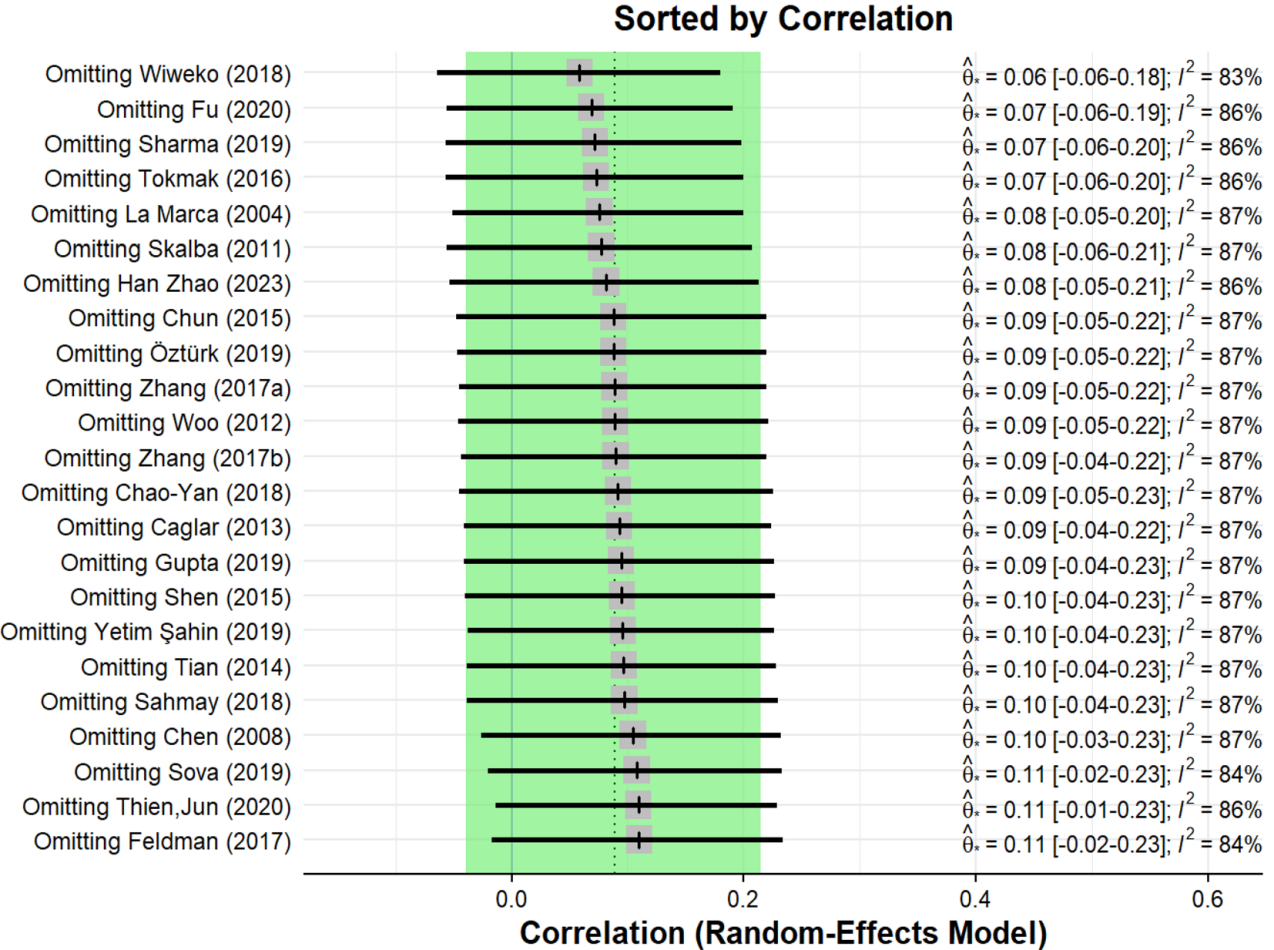
Leave-one-out influential analysis

To identify the possible sources of heterogeneity in the pooled meta-analysis of the correlation between AMH and IR in patients with PCOS, we conducted a leave-one-out influential analysis. This analysis showed that the overall prevalence was strong and did not depend on a single study (Fig. 4). In patients with PCOS, the pooled correlation between AMH and IR ranged from 0.06 (95% CI: 0.060, 0.180) to 0.11 (95% CI: ?0.020, 0.230).

### Publication bias

**Fig. 5:**
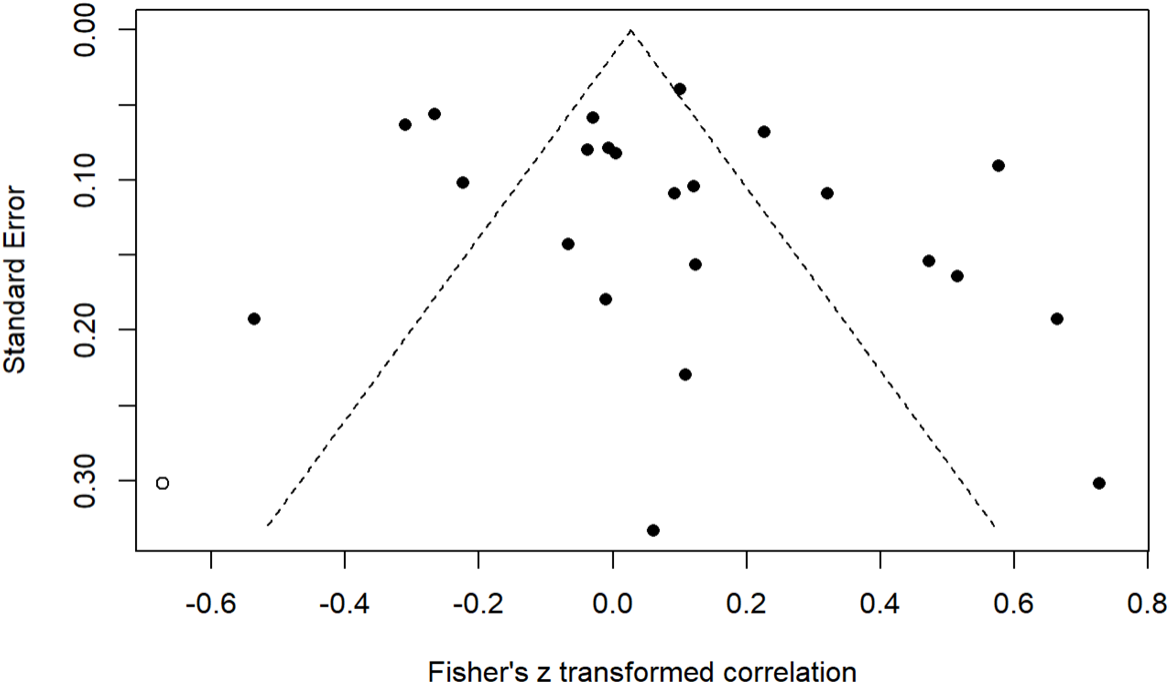
: Funnel plot for assessing publication bias in the included studies

To assess the publication bias of the included studies, we used Begg’s and Egger’s tests. We found no evidence of publication bias in the overall meta-analysis of the correlation between AMH and HOMA-IR in patients with PCOS (Begg’s test, *p* = .177; Egger’s test, *p* = .216). The symmetry of the funnel plot was in agreement with the results of Egger’s tests (Fig. 5). We searched unpublished or gray literature using Google scholar and a web-based search to reduce publication bias.

## Discussion

Although many studies have been conducted regarding the relationship between AMH and IR in PCOS, the findings are conflicting. In this SRMA, we identified a weak correlation between serum AMH and HOMA-IR in patients with PCOS. It is known that both parameters play an important role in the pathophysiology of the disease. However, the results of our SRMA led us to conclude that changes in AMH levels have no significant influence on IR in patients with PCOS. This means that no reduction in AMH level will improve IR in patients with PCOS. Similarly, treating IR will not change AMH levels in PCOS.

We observed no significant variation in the pooled correlation estimate when we conducted subgroup analyses according to region, although different regions may have various genetic and environmental factors that could affect AMH levels [44–46]. In Europe, the subgroup analysis revealed a slightly lower pooled effect estimate (0.099 [95% CI: 0.147, 0.333]) compared with Asia (0.116 [95% CI: −0.050, 0.277]). This subgroup analysis should be judged with caution because of the small number of studies from Asia (*n* = 13) and Europe (*n* = 8). Because only one study was conducted in North America, we could not compare the effect estimates with the North American region. Subgroup analysis by BMI and phenotypes may provide valuable data for the study, as different PCOS phenotypes and BMI have been reported to have different degrees of IR incidence [47]. Previous studies have shown that PCOS patients of hyperandrogenic phenotypes were prone to develop IR compared to the other phenotypes [48, 49]. In turn, IR and excessive BMI may exacerbate the symptoms of hyperandrogenism [50]. However, we could not compare the correlation estimates between different PCOS phenotypes and different classifications of BMI because of limited data.

Among the studies included in the review, we found that various cutoffs for HOMA-IR were used as IR indicators. One study used a cutoff value of > 3.0 [6], and other studies used cutoff values of > 2.5 and > 2.14, based on their population HOMA-IR cutoffs [23, 28]. The variability in HOMA-IR cutoffs may reflect different correlations between AMH and HOMA-IR in patients with PCOS across the studies, as a lower HOMA-IR cutoff will include more subjects with PCOS diagnosed as IR as compared with different studies using higher HOMA-IR values [46].

We acknowledge the limitations of this SRMA. Despite a thorough search strategy, some studies might not have been included. Because of the limited number of studies, it was not possible to assess publication bias across demographic, metabolic, and endocrine parameters, which limited our ability to perform subgroup analyses and attenuated the power of the analyses. A further limitation of this study is the lack of a standardized scale to assess the quality of the included studies. We found statistically significant heterogeneity in most analyses (approximately 80%). This limitation, which has been observed in other meta-analyses of epidemiological studies, may result from unreported factors. By using a random effect model for statistical interpretation, the findings will not be affected by the high degree of heterogeneity, and reliable and more efficient estimates are provided when there is a high degree of heterogeneity [51, 52]. The other causes of potential biases across the studies were possibly the different sample sizes and anthropometry of the study subjects [53].

We could not examine the heterogeneity effect of different age groups in this SRMA because all studies involved young adults. None of the studies were race specific, which leaves room for this variation to be examined.

## Conclusion

To the best of our knowledge, this is the first SRMA to examine the correlation between AMH and IR in patients with PCOS. Our SRMA suggests there is limited or no evidence that high serum AMH levels in patients with PCOS are causally linked to the development of IR. A high level of heterogeneity was potentially caused by different PCOS phenotypes, different BMI classifications, variation in environmental factors and genetics across regions, and different age groups. Subgroup analysis of these factors may reduce the degree of heterogeneity. Future studies on the relationship between AMH and IR in PCOS with alternative interventions may be needed to enhance our understanding.

## Data Availability

The data for this meta-analysis were retrieved from published articles and are available from the author upon request.

## Abbreviations

AMH: Anti-Müllerian hormone
CI: Confidence interval
ECLIA: Electrochemiluminescence immunoassay
ELISA: Enzyme-linked immunosorbent assay
HA: Hyperandrogenism
HOMA-IR: Homeostatic model assessment for insulin resistance
IR: Insulin resistance
JBI: Joanna Briggs Institute
OA: Oligomenorrhea
PCOM: Polycystic ovarian morphology
PCOS: Polycystic ovarian syndrome
PRISMA: Preferred Reporting Items for Systematic Reviews and Meta-analysis
SRMA: Systematic review and meta-analysis
